# Isoprostanes in Veterinary Medicine: Beyond a Biomarker

**DOI:** 10.3390/antiox10020145

**Published:** 2021-01-20

**Authors:** Ashley K. Putman, G. Andres Contreras, Lorraine M. Sordillo

**Affiliations:** Department of Large Animal Clinical Sciences, College of Veterinary Medicine, Michigan State University, 784 Wilson Road, East Lansing, MI 48823, USA; putmanas@msu.edu (A.K.P.); contre28@msu.edu (G.A.C.)

**Keywords:** isoprostane, oxidative stress, lipid peroxidation, veterinary medicine

## Abstract

Oxidative stress has been associated with many pathologies, in both human and animal medicine. Damage to tissue components such as lipids is a defining feature of oxidative stress and can lead to the generation of many oxidized products, including isoprostanes (IsoP). First recognized in the early 1990s, IsoP are formed in numerous biological fluids and tissues, chemically stable, and easily measured by noninvasive means. Additionally, IsoP are highly specific indicators of lipid peroxidation and thereby are regarded as excellent biomarkers of oxidative stress. Although there have been many advancements in the detection and use of IsoP as a biomarker, there is still a paucity of knowledge regarding the biological activity of these molecules and their potential roles in pathology of oxidative stress. Furthermore, the use of IsoP has been limited in veterinary species thus far and represents an avenue of opportunity for clinical applications in veterinary practice. Examples of clinical applications of IsoP in veterinary medicine include use as a novel biomarker to guide treatment recommendations or as a target to mitigate inflammatory processes. This review will discuss the history, biosynthesis, measurement, use as a biomarker, and biological action of IsoP, particularly in the context of veterinary medicine.

## 1. Introduction

Oxidative stress, the imbalance between oxidants and antioxidants leading to tissue damage, has been associated with many diseases in both humans and animals [1]. Oxidants are chemically reactive molecules that are responsible for the tissue damage associated with oxidative stress and are composed of reactive oxygen species (ROS) and reactive nitrogen species (RNS). Produced in moderate amounts as a byproduct of energy generation, oxidants are used in numerous cell signaling and immune pathways under physiologic conditions [2]. Antioxidants are designed to counter oxidants and maintain homeostasis, contributing to what is termed redox balance. However, a disruption in the balance that favors excessive oxidant accumulation leads to oxidative stress [2]. Examples of human diseases with an oxidative stress-related component in their pathophysiology include Alzheimer’s disease, type II diabetes, and heart failure [3,4,5]. In veterinary species, oxidative stress has been associated with similar disorders such as canine counterpart of senile dementia of the Alzheimer type, metabolic stress in dairy cattle, and congestive heart failure in dogs [6,7,8]. Despite the evidence supporting oxidative stress as an important contributor to numerous pathologies, no clinical signs of the process are displayed [9]. Furthermore, it can be challenging to distinguish between redox balance and unchecked oxidants, thereby making it necessary to use specific measurements to determine if oxidative stress is occurring. In fact, the need for reliable oxidative stress biomarkers and an understanding of any physiologic roles they may play in disease pathogenesis has become a priority in the research community.

Broadly, a biomarker can be described as “a defined characteristic that is measured as an indicator of normal biological processes, pathogenic processes or responses to an exposure or intervention” [10]. Several biomarkers of oxidative stress can be identified due to the tissue damage that occurs as a function of increased interactions between ROS and biological molecules. Common biomarkers generated from the reactions between ROS and nucleic acids, proteins, and lipids include 8-hydroxydeoxyguanosine, protein carbonyls, and isoprostanes (IsoP), respectively [11,12]. However, the most favorable biomarkers must be more than merely measurable; they should also show specificity for a particular process, have prognostic value, or correlate with pathology [13].

Since their discovery in the early 1990s, IsoP have become one of the most widely used biomarkers of in vivo oxidative stress because they are highly sensitive and specific, can be measured noninvasively from numerous biological tissues and fluids, and are chemically stable [14,15]. In addition to IsoP themselves, IsoP metabolites are also valuable biomarkers. The metabolite 2,3-dinor-5, 6-dihydro-8-iso-prostaglandin F_2α_, for instance, is only generated in the liver from plasma IsoP and has a longer half-life than its parent compound. Therefore, its presence is considered an accurate representation of systemic oxidative stress over time [16]. Indeed, increased IsoP and IsoP metabolites have been associated with many human oxidative stress-related pathologies, including metabolic syndrome and cardiovascular disease [17]. However, while IsoP continuously become more popular in human medicine, studies regarding their potential role in disease pathogenesis remain sparse. Furthermore, literature of IsoP in veterinary species is relatively limited. This review will discuss the history, biosynthesis, and detection of IsoP, followed by current use and knowledge gaps regarding their role in veterinary medicine. Finally, the review will conclude with thoughts on future considerations for IsoP to be used successfully in veterinary medicine.

## 2. History

Isoprostanes are a relatively new oxidative stress biomarker, having only been characterized in vivo in the last 30 years. However, the earliest evidence of IsoP formation surfaced from in vitro work completed by Pryor and Stanley in the 1970s. They found that when methyl linolenate was autoxidized, a bicyclic endoperoxide was formed as a precursor to prostaglandin-like compounds [18]. In the 1980s, it was shown that the lower side chains of prostaglandin D_2_ (PGD_2_) will isomerize in aqueous solutions to form isomers of 9α,11β-PGF_2α_ when reduced by 11-ketoreductase [19]. Later attempts to describe the presence of these compounds in vivo lead to the discovery of what are now termed isoprostanes [20]. In the early 1990s, Morrow and colleagues used mass spectrometry to characterize the 9α,11β-PGF_2α_ isomers discovered by Wendelborn et al. [19]. They were prompted by the fact that the peaks generated by the 9α,11β-PGF_2α_ isomers increased around 50-fold when the plasma was analyzed after several months of storage at −20 °C when compared to the plasma that was analyzed immediately after collection [21]. Furthermore, the authors found that the addition of antioxidants to the plasma samples would reduce the formation of F-ring prostaglandin-like compounds. This suggested that the molecules were generated independently of enzymatic pathways. First was the discovery of prostaglandin (PG) F_2_-like compounds, termed the F_2_-isoprostanes [21]. Shortly thereafter, Morrow et al. determined that PGE- and PGD-isoprostanes could be generated in vivo as well [22].

Although these earliest discovered IsoP were derived from arachidonic acid, it was soon determined that other polyunsaturated fatty acids could also generate IsoP in the face of interactions with free radicals. Later in the 90s, the omega-3 fatty acids eicosapentaenoic acid (EPA) and docosahexaenoic acid (DHA) were found to produce F_3_-IsoP and F_4_-neuroprostanes, respectively [23,24,25]. Within a decade of the omega-3 IsoP discoveries, the omega-6 fatty acid, adrenic acid, was found to produce its own class of IsoP when undergoing interactions with free radicals [26]. Furthermore, IsoP are not exclusive to animals. Indeed, the plant-based omega-3 fatty acid α-linolenic acid will generate phytoprostanes (PhytoP) [27]. As the name suggests, IsoP are isomers of PG; however, they are synthesized by different mechanisms.

## 3. Biosynthesis

Isoprostanes are formed from interactions between free radicals and plasma membranes. Free radicals are molecules that contain an unpaired electron and are comprised of ROS and RNS [28]. ROS consist of many diverse molecules, including lipid peroxides, hydrogen peroxide, hydroxyl radical (OH^●^), and superoxide anion (O_2_^●−^) [29]. Production of ROS is part of redox signaling in physiological processes in eukaryotes with the majority being formed by the mitochondria during cellular respiration or as a result of nicotinamide adenine dinucleotide phosphate (NADPH) stimulation [29,30]. However, ROS are highly reactive and capable of significant damage to biological molecules such as proteins, DNA, and lipids [30]. For instance, the hydroxyl radical can generate more ROS, alter protein amino acids, and cause lipid peroxidation [31].

**Figure 1:**
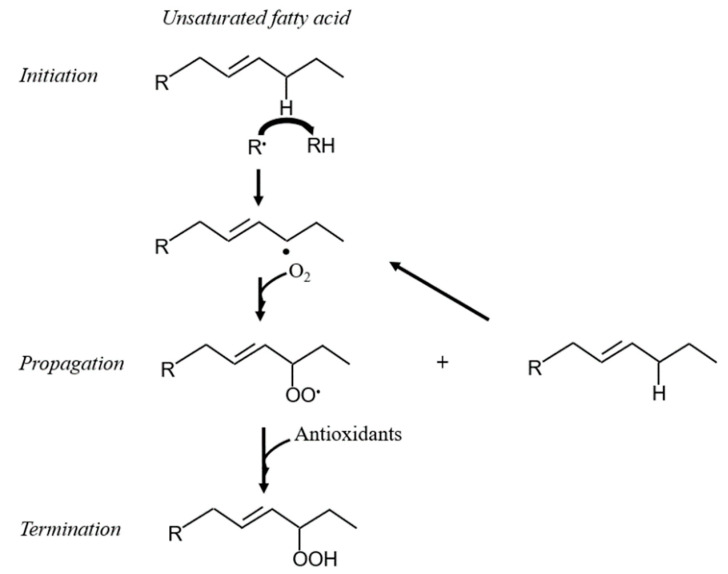
Example of initiation, propagation, and termination pathways during lipid peroxidation. R^•^ = free radical.

**Figure 2:**
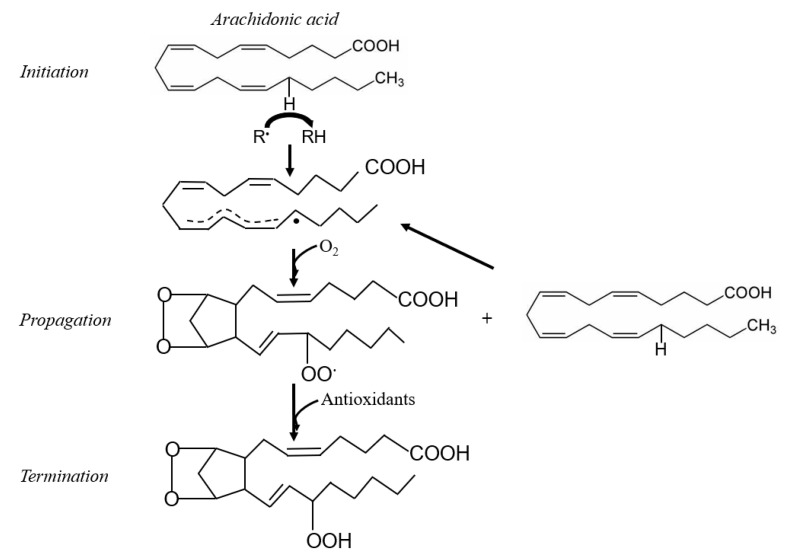
Auto-oxidation of arachidonic acid resulting in the formation of the isoprostane (IsoP) precursor, prostaglandin H (PGH)-like bicyclic endoperoxide. R^•^ = free radical.

**Figure 3:**
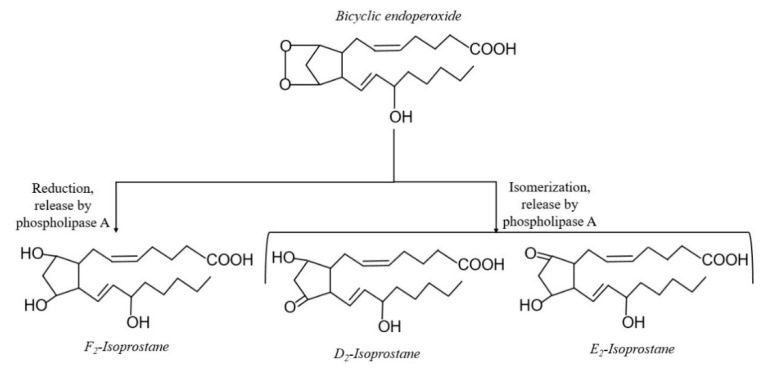
Formation of D-, E-, and F-ring isoprostanes (IsoP) from a prostaglandin H (PGH)-like bicyclic endoperoxide intermediate.

Membrane phospholipids are particularly prone to oxidation [32]. Polyunsaturated fatty acids (PUFA) are a major component of membrane phospholipids, and contain methylene groups flanked by double bonds known as bisallylic groups [33]. Bisallylic groups are readily oxidized because their electrons are pulled to either side by the adjacent double bonds, resulting in weakened bond energies [34]. There are 3 events involved in the free radical-mediated lipid peroxidation chain mechanism: initiation, propagation, and termination (Figure 1) [35]. The first step to IsoP formation involves radical abstraction of a bisallylic hydrogen, or a hydrogen bound to the methylene carbon adjacent to double bonds, from PUFA [36]. The abstraction of the bisallylic hydrogen, which results in the formation of a pentadienyl radical, is followed by the addition of oxygen. At this point, a peroxyl radical is formed, which is capable of propagating more free radical-mediated lipid peroxidation [35]. Sequential rounds consisting of two 5-exo-cyclizations of the peroxyl radical followed by another addition of oxygen and the reduction of the hydroperoxide side chain results in the generation of a bicyclic endoperoxide intermediate that resembles prostaglandin H (PGH) (Figure 2). Due to their instability, the PGH-like bicyclic endoperoxides rapidly react with reducing agents or isomerize to form F-ring IsoP or D- and E-ring IsoP, respectively (Figure 3) [36]. Lipid peroxidation is ceased when antioxidants quench the free radical reactions [35]. 

The initiation step has an equal chance of occurring at any bisallylic hydrogen when multiple bisallylic groups exist in a PUFA. Polyunsaturated fatty acids that contain more bisallylic methylenes have more potential to be oxidized. Therefore, docosahexaenoic acid has the most oxidation potential of the common fatty acids, followed in descending order by eicosapentaenoic acid, arachidonic acid, and finally linoleic acid [35]. Indeed, it has been suggested that docosahexaenoic acid is at an increased susceptibility of oxidative stress in the absence of α-tocopherol and that decreased susceptibility of mitochondrial membranes to oxidative stress may be conferred by the high percentage of linoleic acid in mitochondrial membranes relative to docosahexaenoic acid or eicosapentaenoic acid [37,38]. As all of the biologically important PUFA can be oxidized and any of the available bisallylic hydrogens can be involved in the initiation step, a diverse array of oxidized products can be formed [35]. Primary products of free radical-mediated lipid peroxidation include peroxides and hydroperoxides, which are unstable compounds that often degrade into secondary products [39]. Secondary lipid peroxidation products include aldehydes, ketones, and IsoP [35,39]. Prostaglandins are another lipid peroxidation product that are quite similar to IsoP in structure; however, a few key differences distinguish the two. Generation of IsoP is through a nonenzymatic mechanism, unlike prostaglandin formation which occurs via cyclooxygenase enzymes. In contrast to prostaglandins, in which the PUFA is first released by phospholipase and then oxidized, IsoP are oxidized in situ and then released from the plasma membrane [40]. The pathways of 15-F_2t_-IsoP metabolism are much like that of prostaglandins and involve the rapid formation of β-oxidation products that will ultimately be excreted primarily in the urine [41].

A multitude of regioisomers and stereoisomers of IsoP can theoretically be formed. Considering F_2_-IsoP, 4 regioisomers are generated (5-, 8-, 12-, and 15-series), with the 5-series and 15-series being the most abundant [35]. As the number of bisallylic hydrogens present in a PUFA increases, the number of possible regioisomers and stereoisomers also increases [42]. Indeed, those IsoP derived from DHA, known as F_4_-IsoP or neuroprostanes, can form 8 regioisomers with 128 theoretical stereoisomers [43]. With the rapidly expanding number of IsoP classes, it was pertinent to develop a nomenclature system to distinguish all the molecules. Unfortunately, a consistent nomenclature scheme has been elusive despite 3 major classification systems coming to the forefront by Rokach et al., Taber et al., and Mueller [44,45,46]. The rationale of each system is beyond the scope of this review, but the details are discussed in an article by Mueller [46].

## 4. Measurement Methods

Due to its relevance in many diseases, there is a desire to reliably measure oxidative stress. Logically, it would make sense to measure ROS and antioxidants individually as oxidative stress occurs due to an imbalance of the two. Thorough reviews of both ROS and antioxidants exist in the literature and therefore will not be discussed extensively herein [30,47,48]. Although it is possible to measure ROS and antioxidants to evaluate oxidative stress, these indicators do not provide any information regarding if tissue damage has occurred in the host. Therefore, IsoP have become an important and reliable assessment of oxidative stress because they indicate lipid damage in a sensitive and specific manner [49]. Furthermore, they have been found in all tissues and fluids assessed, which means noninvasive detection is possible [50]. 

As mentioned previously, IsoP are esterified on membrane phospholipids and then subsequently released by phospholipases. Therefore, 2 populations of IsoP exist in any given unaltered sample: one that has been esterified and released (free, unesterified) and one that has been esterified but remains attached to lipids (esterified). The sum of both the free and esterified populations are termed total IsoP, with the majority of IsoP being in the esterified form [51]. Free IsoP are readily measured by many assays but do not account for the IsoP still attached to lipids [52]. In plasma, for instance, esterified IsoP are formed on lipoproteins such as low-density lipoprotein [53]. An increased amount of esterified IsoP in plasma samples despite steady concentrations of free IsoP suggests compartmentalization of lipid peroxidation [54,55]. Additional sample preparation in the form of hydrolysis can be completed to ensure all IsoP are being measured [51].

Biological samples that contain lipids have the potential to undergo peroxidation ex vivo [21]. Therefore, precautions must be taken when assessing IsoP concentrations from these samples. Some considerations for evaluating IsoP concentrations that were not analyzed immediately post-collection include storage conditions. For instance, measuring plasma IsoP concentrations immediately after collection is ideal, but if that is not possible, the use of antioxidants such as butylhydroxytoluene (BHT) can prevent some autoxidation [21]. Indeed, one study found that plasma collected into tubes containing a mixture of 1 mg/mL EDTA, 40 µg/mL BHT, and 1 mg/mL reduced glutathione (GSH) had significantly less oxidation than those collected in EDTA vacutainers. The same study also documented that F_2_-IsoP concentrations in samples stored at −20 °C and −80 °C for 6 months post-collection prior to analysis were significantly elevated compared to those stored at the same temperatures for 1 month. Additionally, the concentrations of F_2_-IsoP were significantly higher in samples stored at −20 °C than those stored at −80 °C, regardless of the amount of time they were stored or if antioxidants were present. One factor that did not seem to significantly alter the concentrations of F_2_-IsoP, however, was if samples were kept at room temperature or 4 °C for up to 4 h prior to sample processing as long as the antioxidant mixture was present [56].

Although IsoP are detectable in all fluids and tissues of mammals, certain samples offer advantages over others. Measuring urine IsoP is advantageous because it is not invasive like other methods, but it is still important to consider that urine IsoP concentrations may not truly reflect systemic IsoP due to rapid clearance and production from the kidney [57]. Recognizing this predicament, Roberts et al. identified the major 15-F_2t_-IsoP metabolite, 2,3-dinor-5, 6-dihydro-8-iso-prostaglandin F_2α_, and found it to be a valuable integrative index of in vivo oxidative stress [58]. As mentioned previously, 2,3-dinor-5, 6-dihydro-8-iso-prostaglandin F_2α_ is more stable than its parent compound and is formed exclusively in the liver, thereby eliminating the concern of confounding contributions from the kidney [16].

Although urine IsoP metabolite may be the most advantageous tool for assessing systemic oxidative stress compared to IsoP in certain circumstances, IsoP still remain as the gold standard in most instances. Mavangira and colleagues investigated the ability of 15-F_2t_-IsoP to be used as a biomarker of oxidative stress in acute bovine coliform mastitis, concluding that free 15-F_2t_-IsoP concentrations in plasma were the most ideal sample because they correlated positively with systemic oxidant status and negatively with glutathione. Moreover, it was determined that free 15-F_2t_-IsoP concentrations in urine and milk were not reflective of systemic or local mammary oxidative stress [55]. This study demonstrated that the most ideal sample for IsoP quantification may not be the most intuitive one. Indeed, it was surprising that free 15-F_2t_-IsoP in milk were inversely correlated with mammary reactive metabolites given that oxidative stress damages mammary tissue and that metabolites are increased during coliform mastitis [55,59]. The unexpected findings in the Mavangira et al. study support the argument that appropriate sample considerations must be taken into account for each individual case [55]. For example, free 15-F_2t_-IsoP were not correlated with milk oxidant status but total milk 15-F_2t_-IsoP were positively correlated, suggesting that hydrolysis may be required for milk samples prior to IsoP quantification [55]. In addition to sample considerations, selection of an appropriate detection method is equally important in IsoP measurement. Two major detection methods available for IsoP measurement include immunological methods and chromatography coupled with mass spectrometry. 

### 4.1. Immunologic Methods

Immunoassays such as enzyme immunoassays and enzyme-linked immunosorbent assays (ELISA) both rely on the basic immunological principle of antibodies binding to a specific antigen [60]. Immunoassays are popular choices when measuring IsoP because they are relatively uncomplicated, relatively inexpensive, and are high-throughput. However, there are many drawbacks to immunoassays used to measure IsoP. One such drawback is that the nature of immunoassays such as ELISA allows for consistent overestimation of IsoP concentrations due to cross-reactivity with related compounds such as alternative IsoP isomers and prostaglandins [61]. In a study comparing gas chromatography-mass spectrometry (GC-MS) and an ELISA method, the ELISA mean and median concentrations of urine 2,3-dinor-5, 6-dihydro-8-iso-prostaglandin F_2α_ were 30-fold higher than those of GC-MS. Furthermore, the Pearson correlation coefficient between ELISA and GC-MS in the study was 0.55, suggesting poor correlation [61]. Similarly, Klawitter and colleagues found that 3 different ELISA kits overestimated the concentration of 15-F_2t_-IsoP in human plasma and urine when compared to tandem liquid chromatography-tandem mass spectrometry (LC/LC-MS/MS). The 3 different ELISA not only had poor agreement with LC/LC-MS/MS, but also with each other, suggesting that results from studies using different assay kits cannot be compared [62]. 

The inconsistent performance of immunoassays also translates to studies conducted in veterinary species. An investigation conducted in dairy cows found that different ELISA kits (Cell Biolabs versus Cayman Chemical) used on the same samples yielded different results. For instance, the Cell Biolabs ELISA kit (San Diego, CA, USA) detected significant differences between free and total 15-F_2t_-IsoP concentrations, while the Cayman Chemical ELISA (Ann Arbor, MI, USA) did not [55]. In dogs, horses, and cattle, 2 separate ELISA assays had very poor correlations to GC-MS and had substantial bias [63]. Thus, while immunoassays represent a convenient option, lack of specificity and consistency in both humans and veterinary species ultimately make them a poor candidate for accurate IsoP measurement. It is, therefore, prudent to interpret results with caution when evaluating literature using immunoassays to measure IsoP concentrations.

### 4.2. Chromatography

Chromatography methods are one of the forerunners of lipid detection and characterization, thereby making them a preferred choice for IsoP measurement [64]. Chromatographic methods are advantageous in that they are capable of sensitive and specific identification of multiple IsoP isomers, even distinguishing both regioisomers and diastereomers [36]. Morrow and Roberts first used GC-MS to quantify urine F_2_-IsoP in the 90s. However, this protocol required the burdensome steps consisting of 2 rounds of thin-layer chromatography along with chemical derivation [65]. Indeed, not only is the multi-step GC process labor-intensive, but it also is complicated by potential contamination, artifact generation, and poor recovery [66]. 

Therefore, much work was invested into improving IsoP detection with chromatography. For example, an assay was developed using liquid chromatography-electrospray ionization (LC-ESI)-MS, where samples were first treated by solid-phase extraction followed by liquid-liquid extraction. With the use of MS/MS, IsoP isomers of the same mass are able to be distinguished based on their specific fragmentation patterns [67]. Sircar and Subbaiah made further improvements on previous attempts and developed a method in 2007 that combined the simplicity of immunoassay preparation with the sensitivity and specificity of LC-MS, along with quantifying total IsoP as opposed to free IsoP only. Indeed, Sircar and Subbaiah’s method required fewer steps than GC-MS, resulting in less sample losses, and was able to be performed at a decreased cost due to use of single quadrupole equipment [66]. 

Finding methods that decrease the expense of chromatography-MS are important as cost is one of the largest limitations [68]. Adding to the expense of chromatographic methods is the expanding number of IsoP isomers to be measured in each sample, provoking the need for distinguishing multiple IsoP isomers during a single run. Rund et al. developed a method to accomplish this task, resulting in the ability to separate 25 F-ring IsoP, 2 PhytoP, and 8 isofurans (IsoF) derived from multiple PUFA precursors in a single sample [36]. Through the constant quest to improve IsoP quantification methods, LC-MS has become the method of choice because it circumvents the variability of immunoassays and the tedious derivatization steps of GC-MS [69,70]. Indeed, LC-MS/MS has relatively short run times, is sensitive and specific, and is capable of detecting very low concentrations of IsoP when using non-radioactive isotope-labeled standards [69]. Development of improved detection methods has contributed to the widespread use of IsoP in the scientific community.

## 5. Use and Physiological Roles

### 5.1. Biomarkers of Lipid Peroxidation

An ongoing challenge in veterinary medicine lies in the ability to diagnose disease promptly so that best management and treatment recommendations can be made. Isoprostanes may be a valuable tool to clinical practice in this capacity because current literature regarding the use of IsoP is largely based on their advantageous nature as biomarkers of oxidative stress-related disorders. At this time, a handful of literature can be found associating a subset of relevant veterinary diseases with altered IsoP concentrations affecting major domestic species [8,55,71,72,73,74]. These studies provide important insights as there is a dearth of information regarding threshold IsoP concentrations that may distinguish between health and disease. Mavangira et al. found that plasma and urine free 15-F_2t_-IsoP were higher in cows afflicted with coliform mastitis when compared to control cows, while milk 15-F_2t_-IsoP concentrations were lower in coliform mastitis cows as assessed by LC-MS/MS. In contrast, hydrolyzed samples analyzed with a Cell Biolabs ELISA yielded higher total milk 15-F_2t_-IsoP in coliform mastitis cows than in controls [55]. Colic is a common condition of horses that may result in veterinary intervention in the form of medical therapy or surgery. However, the numerous causes of colic present a challenge in terms of finding a single biomarker that can reliably determine which treatment may be most beneficial and predict prognosis in affected horses. Noschka and colleagues set out to determine if IsoP and the IsoP metabolite 2,3-dinor-5, 6-dihydro-8-iso-prostaglandin F_2α_ from urine samples would be suitable prognostic predictors in various causes of equine colic. After normalizing urine IsoP and 2,3-dinor-5, 6-dihydro-8-iso-prostaglandin F_2α_ concentrations to urine creatinine, the authors found that both increased in horses with colic compared to healthy control horses. Furthermore, urine 2,3-dinor-5, 6-dihydro-8-iso-prostaglandin F_2α_ concentrations were significantly higher in horses that underwent surgical correction and those that did not survive. Therefore, the authors proposed that 2,3-dinor-5, 6-dihydro-8-iso-prostaglandin F_2α_ may be a useful biomarker for determining if a horse with colic will require surgery or will be less likely to survive [72]. Concentrations of urine IsoP were predictive in a different way. Urine IsoP concentrations were significantly higher in medical colic cases compared to control horses, but surgical cases were not significantly different from controls nor medical colic cases. Additionally, urine IsoP concentrations were significantly higher in horses that survived compared to control, while no difference was detected between nonsurvivors and controls or survivors [72].

Small animals are afflicted with a variety of oxidative stress-related disorders, presenting an opportunity for use of IsoP. Viviano and Van der Wielen supported that dogs with systemic illness undergo more oxidative stress than healthy controls through the use of biomarkers such as urine F_2_-IsoP concentrations [73]. Spinal cord and cardiovascular disease, common conditions seen in veterinary medicine, are associated with oxidative damage [71]. Dogs with intervertebral disk disease undergoing surgery had increased urine F_2_-IsoP: creatinine ratios compared to healthy dogs undergoing surgery, both before and after the surgical procedure. Interestingly, dogs that had more severe neurological signs as a result of their disc disease had lower urine F_2_-IsoP concentrations than those with less severe neurologic signs [71]. Dogs with congestive heart failure had significantly higher 15-F_2t_-IsoP than healthy controls [8]. Chronic kidney disease is also a common ailment in small animals, but can be difficult to diagnose in its early stages due to lack of a sensitive biomarker that can detect the disease before significant organ damage has occurred [75]. Furthermore, oxidative stress has been implicated in the progression of human chronic kidney disease, but had not been thoroughly investigated in cats prior to a study completed by Whitehouse and colleagues [74]. Cats with stage 1 chronic kidney disease had significantly higher urine F_2_-IsoP concentrations than cats in stages 2-4. In contrast to the increased concentrations of IsoP that are associated with more advanced stages of chronic kidney disease in humans, cats in the more severe stages 3 or 4 had lowest urinary IsoP concentrations, even when compared to healthy controls [74]. Differences between human and animal studies illustrate the need to understand species-specific IsoP concentrations so that IsoP may be used effectively in veterinary medicine.

As generation of ROS occurs during normal physiological processes, hence leading to lipid peroxidation, IsoP are also produced routinely in clinically healthy individuals [76]. However, only a few studies describe basal IsoP concentrations in apparently healthy animals. The modern dairy cow undergoes major physiological transitions throughout a typical lactation cycle, especially at the onset and cessation of lactation. For instance, tremendous amounts of energy are required for the copious milk synthesis typical of a modern dairy cow at the onset of lactation, suggesting a necessary increase in ROS production via the mitochondria [77]. A concurrent decrease in antioxidant intake during early lactation can thereby lead to ROS accumulation and lipid peroxidation. Kuhn et al. documented dynamic concentrations of 15-F_2t_-IsoP throughout the lactation cycles of clinically healthy cattle. Indeed, plasma and urine concentrations of the IsoP were significantly lower in mid-lactation cows compared to those in either early or late lactation. However, milk concentrations were significantly lower during early lactation compared to mid- or late lactation [78]. Additional work completed by our lab demonstrated that clinically healthy Holstein cows experience changes in IsoP concentrations throughout the physiological transition from lactating to non-lactating as well. In fact, each of the 7 detected IsoP exhibited its own unique expression profile throughout the transition. Intriguingly, the most well-studied IsoP, 15-F_2t_-IsoP, did not reach statistical significance after adjusting for multiple comparisons, although a relative increase in concentrations was noted for 2 d after abrupt cessation of lactation [79]. Our study demonstrated that other IsoP besides 15-F_2t_-IsoP may be biologically relevant and deserve further study investigating their role in the host. During times of intense exercise, and therefore increased oxygen consumption, Hinchcliff et al. unsurprisingly determined that healthy sled dogs completing 58 km runs on 3 consecutive days had significantly higher plasma concentrations of IsoP than healthy control dogs that did not run at all [80]. Determining the concentrations that may be seen during both disease and health in animals provides a first step in understanding how changes in the concentrations may be impacting the host.

### 5.2. Vascular Regulation

**Figure 4:**
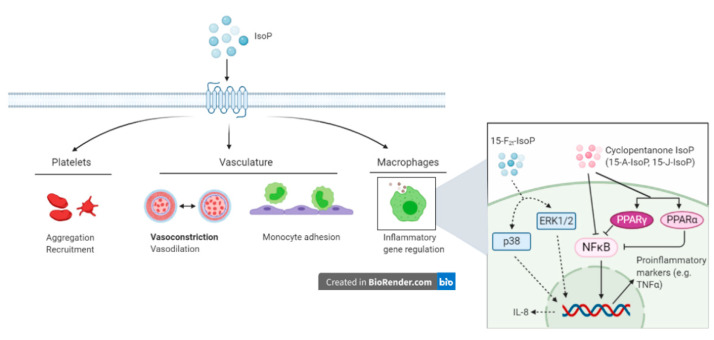
Putative actions of isoprostanes (IsoP) through the thromboxane receptor. Isoprostanes partake in vascular regulation and inflammation through their actions on platelets, the vasculature, and macrophages. During inflammation, IsoP affect monocyte adhesion to the endothelium and alter macrophage inflammatory genes. Insert box: 15-F_2t_-isoprostane modifies monocyte adhesion and increases interleukin-8 production via p38 and ERK1/2. Anti-inflammatory effects of IsoP are mediated by signaling through nuclear factor kappa B and peroxisome proliferator-activated receptor. IsoP = isoprostane; ERK = extracellular-signal-regulated kinase; NFκB = nuclear factor kappa B; PPAR = peroxisome proliferator-activated receptor; IL = interleukin. Created with BioRender.com.

Although IsoP are sensitive and specific biomarkers of oxidative stress, research also is focused on determining potential physiological roles in the host. Vascular endothelium dysfunction is a primary component of oxidative stress and as such, the interaction between IsoP and the vasculature has gathered a considerable amount of research attention [81]. Most of the research supports that a predominant role of IsoP is vasoconstriction, which has been demonstrated in numerous species and tissue types [82]. Early evidence of vasoconstriction was established by directly infusing rat kidneys with 15-F_2t_-IsoP, which revealed a substantial dose-dependent decrease in glomerular filtration rate and renal plasma flow in vivo, both at the whole kidney and single nephron level [83]. Both 15-F_2t_-IsoP and 15-E_2t_-IsoP were shown to cause sustained and dose-dependent coronary vasoconstriction in isolated guinea pig hearts, sometimes decreasing coronary flow by 50% [84]. Vasoconstriction can be strongly attenuated by infusing or treating cells concomitantly with a thromboxane A2 receptor (TP) antagonist (SQ29548), suggesting that IsoP exert their action through TP [83,84]. In fact, numerous studies have supported IsoP as a ligand for TP and Figure 4 summarizes proposed biological consequences as a result of IsoP-TP interaction [85,86]. Isoprostanes are also capable of vasodilation, although this biological effect is often eclipsed by vasoconstriction and is dependent on many factors including the IsoP isomer, concentration, species, vascular bed, and whether or not a ligand is bound to TP [82,87]. For example, 15-E_2t_-IsoP relaxed porcine pulmonary and coronary arteries that had been pre-treated with the TP agonist U46619. Conversely, none of the 6 IsoP tested (including 15-E_2t_-IsoP) relaxed pulmonary veins pre-constricted by U46619 [87]. Table 1 summarizes the effects of IsoP in various tissues and species.

Although vascular tone is one aspect that TP modulate, platelet function regulation is another. Through their action on TP, IsoP impact platelet function in a complex way. For instance, early studies indicated that 15-F_2t_-IsoP increased platelet adhesion in a dose-dependent manner, an effect that could be significantly reduced in the presence of a TP antagonist [90]. Another study conducted by Cranshaw and colleagues found that out of 11 studied IsoP (including 15-F_2t_-IsoP), none induced platelet aggregation in human whole blood on their own. However, the pro-aggregatory responses induced by the TP agonist U46619 were inhibited by 15-F_2t_-IsoP, 8-isoPGE_1_, 15-E_2t_-IsoP, and 8-isoPGF_3α_ in the same study [91]. A more recent study indicated that platelets produce 15-F_2t_-IsoP in the presence of free radicals and that 15-F_2t_-IsoP was important for platelet recruitment [92]. Therefore, IsoP appear to play an important role in mediation of vascular effects, at least in part through actions on TP (Figure 4). 

The TP is a G-protein-coupled receptor distributed widely throughout the body and among various cells, having been described in platelets, endothelial cells, and smooth muscle cells, along with others [93]. Ligand binding to TP can result in coupling to a wide range of G proteins, ultimately leading to downstream effects that impact coagulation, inflammation, and oxidative stress [94,95,96]. For instance, oxidative stress was mitigated when animals were treated with the TP antagonist S18886 as demonstrated by decreased urinary 15-F_2t_-IsoP and evidence of renal pathology in a murine model of diabetic nephropathy [94]. The diversity of TP provides a possible explanation for the divergent effects of IsoP on the vasculature between different species and tissue beds. For instance, work performed by Ogletree and Allen questioned whether both intraspecies and interspecies differences existed between TP of artery, vein, and airway smooth muscles. Although TP appeared to be the same within a species, TP antagonists had significantly divergent potencies between the smooth muscles of rats and guinea pigs [97]. From this early study, the authors concluded that there may not be different subtypes of TP, but there is likely significant variation between species. However, more recent studies have supported the existence of TP heterogeneity. This is exemplified in humans, where there are 2 subtypes of TP commonly termed TP_α_ (placental) and TP_β_ (endothelial) [98]. Although these subtypes are encoded by a single gene, their cytoplasmic tails are alternatively spliced, they are expressed to varying degrees in tissues, and have shown to exert differential signaling and downstream effects [93,99]. For instance, TP_α_ is down-regulated in fibroblasts that have prolonged stimulation with a TP agonist while TP_β_ is up-regulated [99]. It is not true, however, that every species expresses 2 subtypes of TP. In fact, cattle express only one whose amino acid sequence most closely resembles that of the human TP_α_ isoform [100]. Investigating IsoP-TP interaction and the downstream effects in each species represents an area of opportunity to gain a more thorough understanding of how IsoP exert biological actions.

### 5.3. Inflammatory Effects

Outside the scope of vascular regulation, a limited number of studies have investigated other biological roles of IsoP. The intimate relationship between inflammation and oxidative stress suggests that IsoP may serve as an inflammatory mediator. Indeed, 15-F_2t_-IsoP is capable of inhibiting monocyte adhesion to human dermal microvascular endothelial cells, with maximal inhibition being noted at a concentration of 1 µM. However, in human umbilical vein endothelial cells, 15-F_2t_-IsoP enhanced monocyte adhesion at 10^−10^ to 10^−8^ M [101]. To characterize the mechanism by which 15-F_2t_-IsoP caused the suppression of monocyte adhesion to endothelial cells, the authors investigated several direct and indirect effects the IsoP may have. Of the proposed mechanisms, 15-F_2t_-IsoP inhibited monocyte adhesion induced by tumor necrosis factor alpha (TNF_α_), had inhibitory effects comparable to those seen with a TP agonist, and appeared to be working through the p38 and c-Jun N-terminal kinases (JNK) pathway via a TP-mediated mechanism. Furthermore, 15-F_2t_-IsoP appears to encourage the production of a secondary inhibitor of monocyte adhesion, which acts in a TP-independent manner [101]. Scholz and coworkers discovered that 8-isoP also increase interleukin-8 expression in human macrophages [102]. Cyclopentenone IsoP (15-A_2_-IsoP and 15-J_2_-IsoP) are also capable of modulating macrophage inflammatory actions. The cyclopentenone IsoP abrogated inflammatory responses in both RAW 267.4 murine macrophages and primary macrophages via blocking translocation of nuclear factor kappa B (NFκB) to the nucleus. Downstream effects of this blockade included inhibition of nitrite production, which was abrogated by performing glutathione adduction [103]. Interestingly, F_2_-IsoP does not inhibit nitrite production, which implicates the cyclopentenone moiety as being responsible for the anti-inflammatory responses. The cyclopentenone IsoP diverge in their actions under some circumstances though, highlighted by the potent activation of PPARγ by 15-J_2_-IsoP but not 15-A_2_-IsoP [103]. Additional evidence for the anti-inflammatory effects of IsoP has been offered for 15-A_3t_-IsoP, 14-A_4t_-NeuroP, and 4-F_4t_-NeuroP [104,105]. Given the impact IsoP have demonstrated in macrophages, it would likely be beneficial to investigate their effects on other cells that participate in the inflammatory response, such as other leukocytes and endothelial cells.

## 6. Conclusions and Future Directions

Oxidative stress continues to be a cornerstone of numerous disorders in both humans and animals. Lipids readily succumb to oxidation damage, thereby introducing IsoP as the gold standard measurement of oxidative stress. In the last 30 years, studies have established the mechanism of IsoP formation and several measurement methods, along with building some evidence for their physiological role in the host. Large deficits remain, however, in maximizing the use of IsoP in veterinary medicine. For instance, although sufficient methods exist to do so, detecting the presence and concentrations of IsoP in a patient will be of little benefit if there is not an understanding of threshold concentrations that indicate disease or an understanding of how altered IsoP concentrations may be impacting the host. 

To use IsoP most-effectively in veterinary medicine, it will be critical to first establish IsoP concentrations in healthy animals so that a basis for disease detection can occur. Then, measurement of IsoP during various relevant diseases can be performed to elucidate conditions in which IsoP may indicate a disease process. Concurrently, it will also be necessary to determine which sample and processing method is most ideal for each case. In association with changing IsoP concentrations, whether during health or disease, many opportunities remain in determining actions of IsoP in the host. Additionally, it is prudent to perform species-specific studies as IsoP have proven to be complex and incredible amounts of variation have been demonstrated between humans and numerous domestic species. Ultimately, however, learning how IsoP actions benefit or harm the host will be of little use in veterinary medicine without the development of reliable, convenient assays that do not depend on complicated sample preparation or expensive laboratory equipment. If these conditions can be improved upon, both human and veterinary species stand to reap the benefits of a highly sensitive and specific oxidative stress biomarker capable of exerting important, complex physiological effects.

## Figures and Tables

**Table 1:** Putative roles of IsoP on the vasculature of veterinary species from selected literature.

Tissue	Species	IsoP	Biological Effect	Source
Kidney	Rat	15-F_2t_-IsoP	Decreased glomerular filtration rate and renal plasma flow (vasoconstriction)	[83]
Lung	Pig	15-E_2t_-IsoP	Vasodilation when vessels precontracted with a thromboxane receptor agonist; vasoconstriction when precontracted with endothelin-1	[87]
		8-iso-PGE_1_ and 15-F_2t_-IsoP	Modest vasodilation when vessels precontracted	[87]
	Chicken	15-F_2t_-IsoP and 15-E_2t_-IsoP	Vasoconstriction; vasodilation when vessels were precontracted with thromboxane agonist	[88]
Heart	Guinea pig	15-F_2t_-IsoP and 15-E_2t_-IsoP	Vasoconstriction	[84]
	Pig	8-iso-PGE_1_, 15-E_2t_-IsoP, and 15-F_2t_-IsoP	Vasodilation when precontracted with thromboxane receptor agonist	[87]
		15-F_2t_-IsoP	Vasoconstriction	[89]
	Cow	15-F_2t_-IsoP	Vasoconstriction	[89]
Ductus arteriosis; femoral artery	Chicken	15-F_2t_-IsoP and 15-E_2t_-IsoP	Vasoconstriction; vasodilation when vessels were precontracted with thromboxane agonist	[88]
